# A Fully Human Monoclonal Antibody Targeting cKIT Is a Potent Inhibitor of Pathological Choroidal Neovascularization in Mice

**DOI:** 10.3390/pharmaceutics13081308

**Published:** 2021-08-20

**Authors:** Songyi Seo, Koung Li Kim, Yeongju Yeo, Ryul-I Kim, Hayoung Jeong, Jin-Ock Kim, Sun-Hwa Song, Mi-Jin An, Jung-Woong Kim, Hye Kyoung Hong, Min Hee Ham, Se Joon Woo, Jong-Hyuk Sung, Sang Gyu Park, Wonhee Suh

**Affiliations:** 1 Department of Global Innovative Drug, Graduate School of Chung-Ang University, Seoul 06974, Korea; ssi4053@naver.com (S.S.); mh8541@naver.com (Y.Y.); ous3@naver.com (R.-I.K.); gkdud8160@naver.com (H.J.); 2 College of Pharmacy, Chung-Ang University, Seoul 06974, Korea; crux777@hanmail.net; 3 College of Pharmacy, Ajou University, Suwon 16499, Korea; kjo8909@ajou.ac.kr (J.-O.K.); thdtjsghk@daum.net (S.-H.S.); 4 Department of Life Science, Chung-Ang University, Seoul 06974, Korea; dksalwls333@gmail.com (M.-J.A.); jungkim@cau.ac.kr (J.-W.K.); 5 Department of Ophthalmology, Seoul National University College of Medicine, Seoul National University Bundang Hospital, Seongnam 13620, Korea; alpaomega@hanmail.net (H.K.H.); miniham@naver.com (M.H.H.); sejoonwoo@gmail.com (S.J.W.); 6 College of Pharmacy, Yonsei University, Incheon 21983, Korea; brian99@yonsei.ac.kr

**Keywords:** age-related macular degeneration, choroidal neovascularization, cKIT, fully human monoclonal antibody, stem cell factor

## Abstract

Stem cell factor (SCF) and its receptor, cKIT, are novel regulators of pathological neovascularization in the eye, which suggests that inhibition of SCF/cKIT signaling may be a novel pharmacological strategy for treating neovascular age-related macular degeneration (AMD). This study evaluated the therapeutic potential of a newly developed fully human monoclonal antibody targeting cKIT, NN2101, in a murine model of neovascular AMD. In hypoxic human endothelial cells, NN2101 substantially inhibited the SCF-induced increase in angiogenesis and activation of the cKIT signaling pathway. In a murine model of neovascular AMD, intravitreal injection of NN2101 substantially inhibited the SCF/cKIT-mediated choroidal neovascularization (CNV), with efficacy comparable to aflibercept, a vascular endothelial growth factor inhibitor. A combined intravitreal injection of NN2101 and aflibercept resulted in an additive therapeutic effect on CNV. NN2101 neither caused ocular toxicity nor interfered with the early retinal vascular development in mice. Ocular pharmacokinetic analysis in rabbits indicated that NN2101 demonstrated a pharmacokinetic profile suitable for intravitreal injection. These findings provide the first evidence of the potential use of the anti-cKIT blocking antibody, NN2101, as an alternative or additive therapeutic for the treatment of neovascular AMD.

## 1. Introduction

Neovascular age-related macular degeneration (AMD) is a common cause of irreversible vision loss predominantly affecting the elderly population [1]. The pathology of neovascular AMD is characterized by choroidal neovascularization (CNV), where new immature blood vessels exhibit growth from the underlying choriocapillary into the subretinal pigment epithelium or subretinal space [2]. These immature blood vessels present with leakage of fluids and blood into and under the retina, leading to vision loss or distortion. Vascular endothelial growth factor (VEGF) plays a pivotal role in pathological NV during the development of neovascular AMD, and anti-VEGF therapy (such as aflibercept, ranibizumab, and bevacizumab) is considered a standard treatment for neovascular AMD in clinical practice [2,3,4]. However, there have been concerns regarding the potential long-term side effects of anti-VEGF therapy because of the inhibition of physiological VEGF function [5,6,7,8,9,10,11]. Therefore, there is an immense need for identifying previously unrecognized disease targets and for developing novel therapeutics.

We previously identified stem cell factor (SCF) and its receptor, cKIT, as novel targets for neovascular eye diseases [12]. At hypoxia, cKIT was highly expressed in vascular endothelial cells, and it enhanced SCF-mediated angiogenesis through the glycogen synthase kinase (GSK)-3β/β-catenin pathway. The hypoxia-induced increase in SCF/cKIT signaling mediated pathological ocular NV in murine models of neovascular AMD and proliferative diabetic retinopathy (PDR). In addition, there is substantial evidence implicating SCF and cKIT in human neovascular eye diseases [13,14]. The expression of SCF and cKIT is highly upregulated in patients with active PDR compared to that in patients with quiescent PDR or nondiabetic patients [13,14]. These findings suggest that anti-SCF/cKIT therapy is a promising therapeutic strategy for treating neovascular eye diseases.

Recently, NN2101, a fully human monoclonal antibody (IgG) targeting cKIT, was generated and characterized [15]. NN2101 binds to the SCF-binding regions of human cKIT with a dissociation constant (K_D_) in the picomolar range. Western blotting and competitive enzyme-linked immunosorbent assay (ELISA) indicated that NN2101 completely inhibited the binding of cKIT to SCF and blocked the activation of cKIT downstream signaling molecules in human leukemic cells. In addition, NN2101 exhibits high specificity to cKIT, reduced effector function, and low immunogenicity. In this study, we investigated the therapeutic efficacy of NN2101 in a murine model of neovascular AMD and determined the ocular toxicity and pharmacokinetics (PK) of NN2101.

## 2. Materials and Methods

### 2.1. Cell Culture

Human umbilical vein endothelial cells (HUVECs; Lonza, Walkersville, MD, USA), murine endothelial cells (American Type Culture Collection, Manassas, VA, USA), human retinal microvascular endothelial cells (HRMEC; Cell Systems, Kirkland, WA, USA), human ocular choroid fibroblast (HOCF; ScienCell, Carlsbad, CA, USA), human corneal fibroblast (HK; ScienCell, Carlsbad, CA, USA), and human retinal pigmented epithelial cells (ARPE-19; American Type Culture Collection, Manassas, VA, USA) were cultured with appropriate culture media, according to the manufacturer’s protocol. For hypoxic treatment, cells were placed in a modular incubator chamber (MIC-101; Billups-Rothenberg Inc., Del Mar, CA, USA) in an atmosphere consisting of 93% N_2_, 5% CO_2_, and 2% O_2_.

### 2.2. Western Blotting

Cells and tissues were lysed with lysis buffer (Thermo Scientific, Rockford, IL, USA). The proteins were separated by sodium dodecyl sulfate-polyacrylamide gel electrophoresis and then transferred to a nitrocellulose membrane. Nuclear and cytosolic fractions of β-catenin were separated using nuclear and cytoplasmic extraction reagent (Thermo Scientific, Rockford, IL, USA), according to the manufacturer’s instructions. Blots were hybridized with the appropriate primary IgG, followed by incubation with horseradish peroxidase-conjugated secondary IgG. Immunoreactive bands were visualized using a chemiluminescent reagent (Amersham Biosciences, Piscataway, NJ, USA). The IgGs used in the experiment are listed in Supplementary Table S1. Densitometry was performed using ImageJ software (National Institute of Health, Bethesda, MD, USA).

### 2.3. In Vitro Angiogenesis Assay

To analyze the inhibitory effect of NN2101 on SCF-induced in vitro angiogenesis at hypoxia, tube formation, scratch wound migration, and cell proliferation assays were performed as previously described [16]. For the tube formation assay, HUVECs were seeded in 24-well plates coated with Matrigel (Corning, Tewksbury, MA, USA) and were then subjected to treatment with endothelial basal medium (EBM, Lonza, Walkersville, MD, USA) containing 1% fetal bovine serum (FBS, Lonza, Walkersville, MD, USA) or supplemented with recombinant human SCF (rh SCF; R&D Systems, Minneapolis, MN, USA; 50 ng/mL) and/or NN2101 (1 μg/mL) for 6 h. Tube networks were quantified by measuring the tube lengths. For the scratch wound migration assay, confluent monolayers of HUVECs were wounded using pipette tips and incubated for 24 h in EBM containing 1% FBS or supplemented with rh SCF (50 ng/mL) and/or NN2101 (1 μg/mL). Cell migration was assessed by measuring the area of cells that had migrated from the wound edges. For the cell proliferation assay, HUVECs were seeded into 96-well plates and incubated for 2 days in EBM containing 1% FBS or supplemented with rh SCF (50 ng/mL) and/or NN2101 (1 μg/mL). Cell proliferation was quantified using a cell counting kit-8 assay (Dojindo Molecular Technology, Inc., Rockville, MD, USA). In addition, in vitro angiogenesis assays were performed similarly in murine endothelial cells that were subjected to treatment with or without recombinant murine SCF (rm SCF; R&D Systems, Minneapolis, MN, USA; 50 ng/mL) and NN2101 (1 μg/mL).

### 2.4. Animals

Six- to eight-week-old male mice and New Zealand white male rabbits weighing 1.5–2 kg were purchased from Orient Co. (Seoul, Korea). The animals were cared for in accordance with the Guide for the Care and Use of Laboratory Animals published by the United States National Institutes of Health. All animal procedures used in this study were approved by the Institutional Animal Care and Use Committees (IACUC) of Chung-Ang University (IACUC number: 201800044, start date: 4 May 2018) and Seoul National University Bundang Hospital (IACUC number: 2018BA1801-239/003/02, start date: 9 January 2018). For the surgical procedures, mice were anesthetized with an intraperitoneal injection of a mixture of ketamine (100 mg/kg; Yuhan Co., Seoul, Korea) and xylazine (6 mg/kg; Bayer Korea, Ltd., Seoul, Korea). Pupils were dilated using topical drops of phenylephrine and tropicamide (Santen, Osaka, Japan). Rabbits were anesthetized with an intramuscular injection of Zoletil (a mixture of tiletamine and zolazepam; 15 mg/kg; Virbac Laboratories, Carros, France) and xylazine (5 mg/kg) and the pupils were dilated with ophthalmic drops of 1% proparacaine (Alcon Laboratories, Inc., Fort Worth, TX, USA). The level of anesthesia was monitored by toe-pinch reflex. Euthanasia was performed by cervical dislocation following in anesthesia.

### 2.5. Animal Model of Laser-Induced CNV

Laser photocoagulation (wavelength: 532 nm; fixed diameter: 50 μm; intensity: 300 mW; duration: 0.70 s) was induced to create experimental CNV lesions in mice using an image-guided laser system (Micron IV, Phoenix Research Laboratories, Pleasanton, CA, USA), as previously described [17]. Immediately after laser injury, mice received an intravitreal injection of NN2101, aflibercept (Regeneron Pharmaceuticals, Tarrytown, NY, USA), a combination of NN2101 and aflibercept, or phosphate buffered saline (PBS) vehicle. For the quantification of laser-induced CNV lesions, eyes were enucleated 7 days after laser photocoagulation. The posterior eye-cups consisting of the retinal pigment epithelium (RPE), choroid, and sclera were dissected. The dissected RPE-choroid-sclera were subjected to treatment with blocking solution (5% bovine serum albumin, 5% normal goat serum, and 0.5% Triton X-100 in PBS) for 1 h at room temperature and stained with Alexa Fluor^®^ 594-conjugated isolectin Griffonia simplicifolia IB4 (1:50 dilution; Invitrogen, Carlsbad, CA, USA) overnight at 4 °C and flat-mounted onto slides. Images of CNV lesions were obtained using a confocal microscope (Carl Zeiss, Jena, Germany), and the fluorescence intensity of each image was analyzed using ImageJ software (version 1.52, National Institutes of Health, Bethesda, MD, USA) [17]. CNV areas of samples were expressed relative to that of controls.

### 2.6. Combination Index (CI)

CI analysis on dose-response data was performed to classify drug interactions into the categories of synergy, additivity, or antagonism. Dose-response data for NN2101 and aflibercept were plotted and analyzed using GraphPad Prism software (GraphPad Software Inc., San Diego, CA, USA), and the CI value was calculated using the formula proposed by Chou and Talalay [18]. A CI of less than, equal to, or more than 1 indicates antagonism, additivity, or synergy, respectively.

### 2.7. Immunohistochemistry and Terminal Deoxynucleotidyl Transferase dUTP Nick End-Labeling (TUNEL) Assay

Eyes were enucleated, fixed, and embedded in paraffin. After quenching endogenous peroxidase activity and blocking with 10% normal goat serum, tissue sections were stained with anti-glial fibrillary acidic protein (GFAP) IgG and fluorescent secondary IgG. Nuclei were stained with 4′,6-diamidino-2-phenylindole (DAPI; Vector Laboratories, Burlingame, CA, USA). Images were obtained using a confocal microscope. All images shown are representative of >3 independent experiments. TUNEL assays were performed on paraffin sections using the in situ cell death detection kit (Roche Applied Science, Mannheim, Germany). Tissue sections subjected to treatment with DNase I (1500 U/mL, Roche Applied Science) were used as a positive control. Images were obtained using confocal or fluorescence microscope (Olympus, Tokyo, Japan). Six to ten sections were examined per group.

### 2.8. Live and Dead Cell Double Staining Assay

Cells were seeded in 96-well plates and incubated in complete growth medium supplemented with or without NN2101. Three days later, cells were stained with calcein-AM (Sigma-Aldrich, St. Louis, MO, USA; 2 μg/mL), propidium iodide (PI; Sigma-Aldrich; 2 μg/mL), and Hoechst (Sigma-Aldrich; 20 μM) to determine the percentage of live or dead cells using Celigo (Nexelom Bioscience, Lawrence, MA, USA).

### 2.9. Ocular Pharmacokinetics (PK)

Ocular PK analysis was performed as previously described [19]. In brief, NN2101 (600 μg in 30 μL) was intravitreally injected into the right eyes of anesthetized and pupil-dilated rabbits, 1 mm behind the surgical limbus in the superotemporal quadrant using a 30-gauge needle and a Hamilton syringe. Left eyes received no intravitreal injection. Three rabbits were sacrificed at each time point (1 h, 1, 3, 6, 9, 14, and 30 days after injection). The serum was harvested, and both the right and left eyes were enucleated and immediately frozen at −80 °C. The frozen eyes were separated into three parts: vitreous humor, aqueous humor, and retina/choroid. The concentration of NN2101 was measured using an indirect ELISA. The concentration-time data of NN2101 were fit using a standard non-compartmental analysis to determine the PK parameters using WinNonlin software version 6.4 (Certara, Ronceton, NJ, USA).

### 2.10. Retinal Vascular Development

NN2101 was injected intravitreally into mice at postnatal day (P) 2, and retinal blood vessels were analyzed at P6. Radial length of retinal blood vessels was measured as the shortest distance from the optic disc to the peripheral vascular front in each quadrant of the retina. Number of sprouting endothelial cells was measured in 0.02 mm^2^ areas of vascular front in each retina. Vascular density in whole-mounted retina was measured as an IB4-positive area divided by measured total area of the retina; it was presented as a percentage. For each quantification, at least four images per whole-mount retina were obtained and analyzed using ImageJ software or LSM Image software (Carl Zeiss, Jena, Germany).

### 2.11. Statistical Analysis

GraphPad Prism software was used to analyze the data. All data in the article passed the normality and equal variance tests. Statistical significance was evaluated using an unpaired Student’s *t*-test or one-way ANOVA with Bonferroni’s post hoc multiple comparison test. The data are presented as the mean ± SEM with *p*-values < 0.05 considered significant. The number of samples is indicated using *n*.

## 3. Results

### 3.1. The Anti-cKIT Blocking IgG, NN2101, Inhibits SCF/cKIT-Mediated Angiogenic Signaling and Angiogenesis in Human Endothelial Cells at Hypoxia

**Figure 1:**
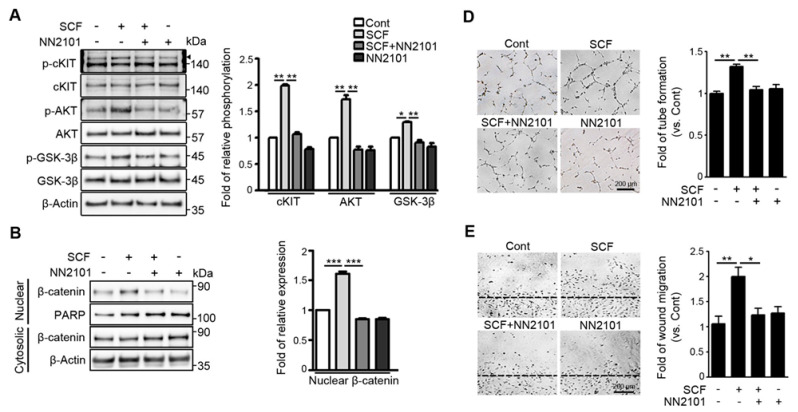

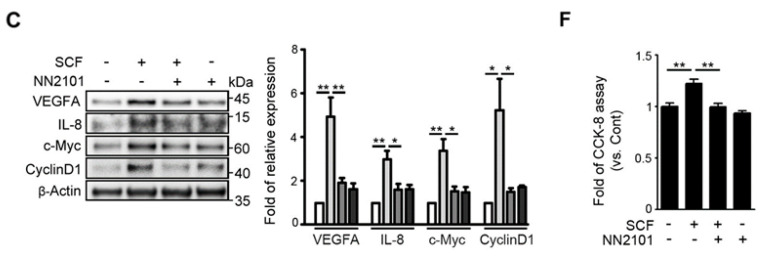
The anti-cKIT blocking IgG, NN2101, inhibits SCF/cKIT-mediated angiogenic signaling and angiogenesis in human endothelial cells at hypoxia. (**A**) NN2101 inhibits the SCF-induced phosphorylation of cKIT, AKT, and GSK-3β in hypoxic endothelial cells. HUVECs cultured at hypoxia were subjected to treatment with or without recombinant human SCF (rh SCF) (50 ng/mL) and NN2101 (5 μg/mL). Phosphorylation of cKIT (p-cKIT; arrowhead), AKT (p-AKT), and GSK-3β (p-GSK-3β; Ser9) was assessed using Western blotting. Band intensities of phosphorylated proteins were normalized to those of total proteins and expressed as the fold increase ± SEM, compared to those in the untreated control group. (set as 1; one-way ANOVA with Bonferroni post hoc multiple comparison test, * *p* < 0.05, ** *p* < 0.001, *n* = 3). (**B**) NN2101 blocks the SCF-induced increase in the nuclear translocation of β-catenin in hypoxic endothelial cells. Nuclear and cytosolic fractions of β-catenin in cells subjected to treatment as described in (**A**) were determined by Western blotting analysis. PARP and β-Actin were used as internal loading controls for nuclear and cytosol fractions, respectively. Band intensities of the nuclear fraction of β-catenin were normalized to the PARP band intensities and expressed as the fold increase ± SEM, compared to those in the untreated control group (set as 1; one-way ANOVA with Bonferroni post hoc multiple comparison test, *** *p* < 0.001, *n* = 3). (**C**) NN2101 blocks the SCF-induced increase in the expression of β-catenin target genes in hypoxic endothelial cells. The protein levels of VEGFA, IL-8, c-Myc, and CyclinD1 were assessed as described in (**A**) (one-way ANOVA with Bonferroni post hoc multiple comparison test, * *p* < 0.05, ** *p* < 0.01, *n* = 3). (**D**–**F**) NN2101 suppresses the SCF-induced angiogenesis in hypoxic endothelial cells. Tube formation (**D**), scratch wound migration (**E**), and cell proliferation assays (**F**) were performed with HUVECs subjected to treatment with or without rh SCF (50 ng/mL) and NN2101 (1 μg/mL) at hypoxia. Tube formation and scratch wound migration were quantified by measuring the tube length and relative area covered by cells that had migrated from the wound edges, respectively. Cell proliferation was determined using a cell counting kit-8 assay. The data are expressed as the fold increase ± SEM compared to that in the untreated control group (Cont) (one-way ANOVA with Bonferroni post hoc multiple comparison test, * *p* < 0.05, ** *p* < 0.01, *n* > 4). Scale bars = 200 μm.

Our previous study showed that, in hypoxic endothelial cells, SCF/cKIT signaling enhances angiogenesis by activating the GSK-3β/β-catenin pathway [12]. To investigate whether the anti-cKIT IgG, NN2101, blocked the activation of the SCF/cKIT signaling pathway, we performed Western blotting and found that treatment with NN2101 substantially inhibited the SCF-mediated phosphorylation of cKIT, AKT, and GSK-3β in hypoxic human endothelial cells (Figure 1A). The phosphorylation of GSK-3β induces the phosphorylation and nuclear translocation of β-catenin, leading to the transcription of β-catenin target genes (Figure 1B,C). Therefore, the treatment with NN2101 abrogated the SCF-induced increase in the nuclear fraction of β-catenin and the protein expression of β-catenin target genes related to angiogenesis (VEGFA, IL-8, c-Myc, and CyclinD1) in hypoxic human endothelial cells (Figure 1B,C). Moreover, the treatment of human endothelial cells with NN2101 completely inhibited the SCF-induced increase in tube formation, scratch wound migration, and cell proliferation at hypoxia (Figure 1D–F).

### 3.2. Intravitreal Administration of NN2101 Suppresses SCF/cKIT Signaling and the Pathological CNV in a Murine Model of Neovascular AMD

**Figure 2:**
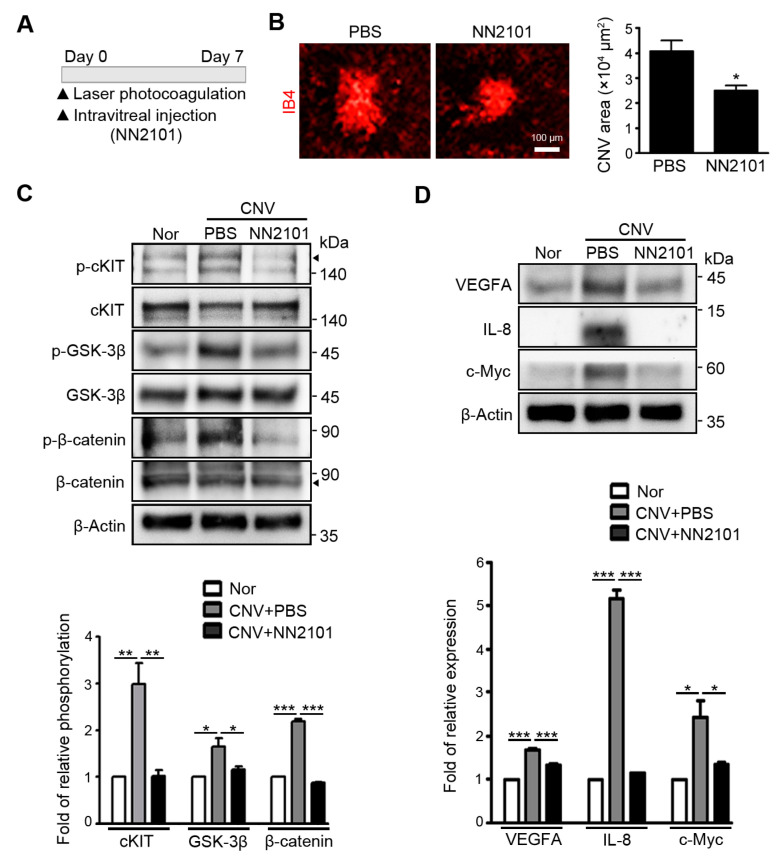
Intravitreal administration of NN2101 suppresses SCF/cKIT signaling and the pathological CNV in murine model of neovascular AMD. (**A**) schematic diagram of laser-induced CNV experiments. Immediately after laser-induced rupture of Bruch’s membrane, mice received an intravitreal injection of NN2101 (1 μg in 1 μL) or an equivalent volume of PBS vehicle. On day 7 after laser photocoagulation, choroidal tissues were harvested for further analysis; (**B**) representative images of flat-mounted choroids with CNV lesions and quantification of CNV lesion area. Choroidal flat mounts were stained with IB4 (red) and the relative CNV area was quantified by measuring the fluorescence intensity of images with IB4-positive areas (unpaired Student’s *t*-test, * *p* < 0.05, *n* > 34 laser spots, *n* > 12 mice per group). Scale bar = 100 μm. (**C**) The phosphorylation of cKIT (p-cKIT; arrowhead), GSK-3β (p-GSK-3β; Ser9), and β-catenin (p-β-catenin; Ser33/37/Thr41) and (**D**) the protein levels of β-catenin target genes (VEGFA, IL-8, and c-Myc) were assessed using Western blotting and are expressed as the fold increase ± SEM compared to those in the normal (Nor) control group (one-way ANOVA with Bonferroni post hoc multiple comparison test, * *p* < 0.05, ** *p* < 0.01, *** *p* < 0.001, *n* = 3). β-Actin was used as a loading control. All data are presented as the mean ± SEM.

NN2101 not only binds strongly to human cKIT (K_D_ = 2.83 × 10^−12^ M), but also binds to mouse cKIT with a high affinity (K_D_ = 11.5 × 10^−9^ M) [15]. Indeed, NN2101 completely blocked the SCF-induced increase in migration and tube formation of murine endothelial cells (Supplementary Figure S1). Therefore, the cross-species reactivity of NN2101 allowed the assessment of the efficacy of NN2101 in vivo. We investigated the therapeutic effect of NN2101 on pathological NV in the choroid using a common murine model of neovascular AMD [17]. Immediately after laser photocoagulation, mice were intravitreally injected with NN2101 or an equivalent volume of PBS vehicle (Figure 2A). Quantitative analysis of CNV lesions revealed that intravitreal administration of NN2101 significantly reduced the area of CNV lesions compared with the vehicle (Figure 2B). The choroidal tissues of NN2101-injected CNV mice exhibited a substantial decrease in the phosphorylation of cKIT, GSK-3β, and β-catenin to levels as low as that in the choroidal tissues of normal mice (Figure 2C). Consistently, protein levels of β-catenin target genes (VEGFA, IL-8, and c-Myc) were markedly reduced in the choroidal tissues of NN2101-injected CNV mice (Figure 2D). These results demonstrate that NN2101 efficiently blocked SCF/cKIT signaling and reduced the pathological CNV in a murine model of neovascular AMD.

### 3.3. A Combination Therapy of NN2101 and Aflibercept Additively Inhibits Pathological CNV in Mice

**Figure 3:**
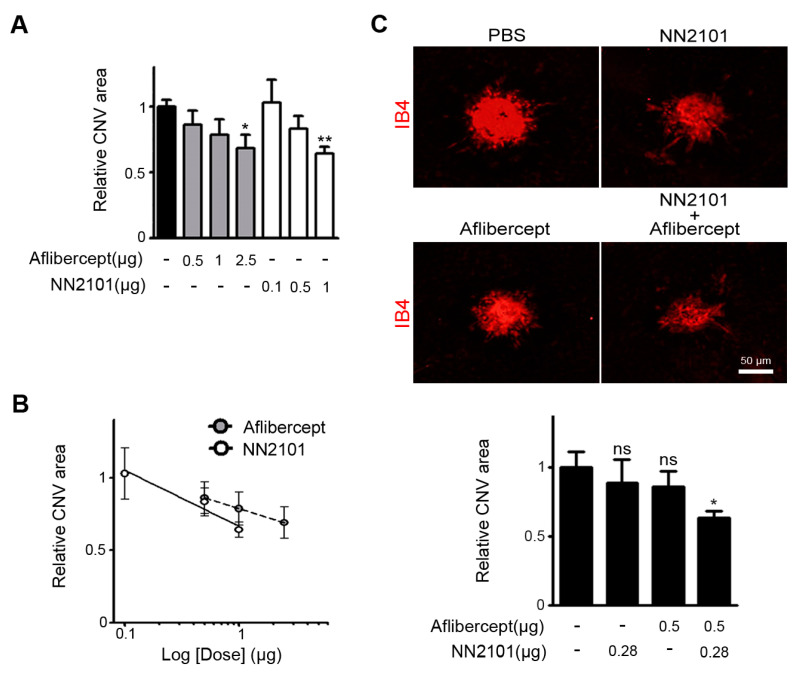
A combination therapy of NN2101 and aflibercept additively inhibits pathological CNV in mice. (**A**) Quantification of CNV lesion area of mice subjected to treatment with an intravitreal injection of PBS or the indicated dose of either NN2101 or aflibercept (one-way ANOVA with Bonferroni post hoc multiple comparison test, * *p* < 0.05, ** *p* < 0.01 vs. PBS, *n* > 17 laser spots, *n* > 6 mice per group); (**B**) dose–response data of NN2101 and aflibercept in (**A**) were plotted with superimposed linear regression lines; (**C**) representative images of flat-mounted choroids with CNV lesions and quantification of CNV lesion area of mice subjected to treatment with an intravitreal injection of NN2101, aflibercept, combination of NN2101 and aflibercept, or PBS (one-way ANOVA with Bonferroni post hoc multiple comparison test, ns = not significant, * *p* < 0.05 vs. PBS, *n* > 21 laser spots, *n* > 7 mice per group). Scale bar = 50 μm. All data are presented as the mean ± SEM.

We next compared the therapeutic efficacies of NN2101 and aflibercept, an anti-VEGF agent used in clinical practice. At a dose of 1 μg, NN2101 significantly reduced the pathological CNV with an efficacy comparable to that of the human equivalent dose (2.5 μg) of aflibercept in mice (Figure 3A). When relative CNV areas were plotted as a function of logarithm of doses, we noted that both NN2101 and aflibercept exhibited a linear correlation between the dose and therapeutic effect (Figure 3B). We then investigated whether a combination of NN2101 and aflibercept would induce a synergistic, additive, or antagonistic effect. We conducted the constant ratio drug combination assay based on the Chou–Talalay CI method [18]. A combination of suboptimal doses of aflibercept (0.5 μg) and NN2101 (0.28 μg) significantly blocked the pathological CNV with an effect comparable to that of 2.5 μg of aflibercept (Figure 3C). Furthermore, the CI value was calculated to be close to 1, which indicated that a combination therapy using NN2101 and aflibercept additively improved the therapeutic efficacy against the pathological CNV.

### 3.4. NN2101 Does Not Induce Ocular Toxicity

**Figure 4:**
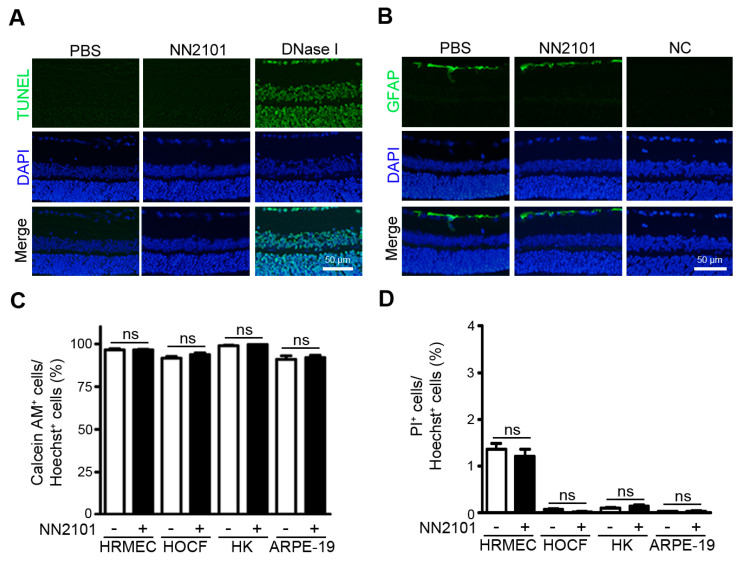
NN2101 does not induce ocular toxicity. (**A**,**B**) an intravitreal injection of NN2101 induces no ocular toxicity in normal adult mice. Mice received a single intravitreal injection of NN2101 (20 μg in 1 μL) or an equivalent volume of PBS. Eyes were harvested two weeks after injection. TUNEL assay (**A**) and immunofluorescence staining with anti-GFAP IgG (**B**) were performed on paraffin sections of the whole eyes. In the TUNEL assay, DNase I-treated sections were included as positive controls. In the immunofluorescence staining with anti-GFAP IgG, sections stained with irrelevant nonspecific IgGs were included as negative controls (NC). The nuclei are shown in blue (DAPI). Representative images in (**A**,**B**) were selected from three independent experiments with similar results. All scale bars = 50 μm. (**C**,**D**) NN2101 induces no cytotoxicity in human ocular cells. HRMECs, HOCF, HK, and APRE-19 cells were incubated with NN2101 (400 μg/mL) or PBS for 3 days, and the percentages of calcein-AM-positive live cells (**C**) and PI-positive dead cells (**D**) were assessed. The data are shown as the mean ± SEM (unpaired Student’s *t*-test, ns = not significant, *n* = 6).

We investigated whether an intravitreal injection of NN2101 induced retinal toxicity in mice. Normal adult mice were intravitreally injected with NN2101 at a dose of 20 μg, which was 20-fold higher than the effective dose (1 μg) in CNV mice. Two weeks after treatment, TUNEL assay and GFAP immunohistochemistry were performed to assess cell apoptosis and retinal tissue stress, respectively. Examination of retinal tissues revealed no difference in cell apoptosis and GFAP expression between the NN2101-injected and control eyes (Figure 4A,B). These results indicate the absence of intraocular toxicity of NN2101 in normal adult mice. In addition, we tested whether NN2101 exerted any antiproliferative or cytotoxic effect in human ocular cells, including retinal endothelial cells, choroidal and corneal fibroblasts, and RPE cells. The live and dead cell double staining assay showed that NN2101 did not impair the cell viability and induced no cytotoxicity in any of the cells subjected to treatment with NN2101 at a concentration of 400 μg/mL, which was 2-fold greater than the vitreous concentration of NN2101 in CNV mice (1 μg NN2101 in 5 μL murine vitreous fluid corresponds to 0.2 mg/mL) (Figure 4C,D).

### 3.5. NN2101 Does Not Affect Early Retinal Vascular Development in Mice

**Figure 5:**
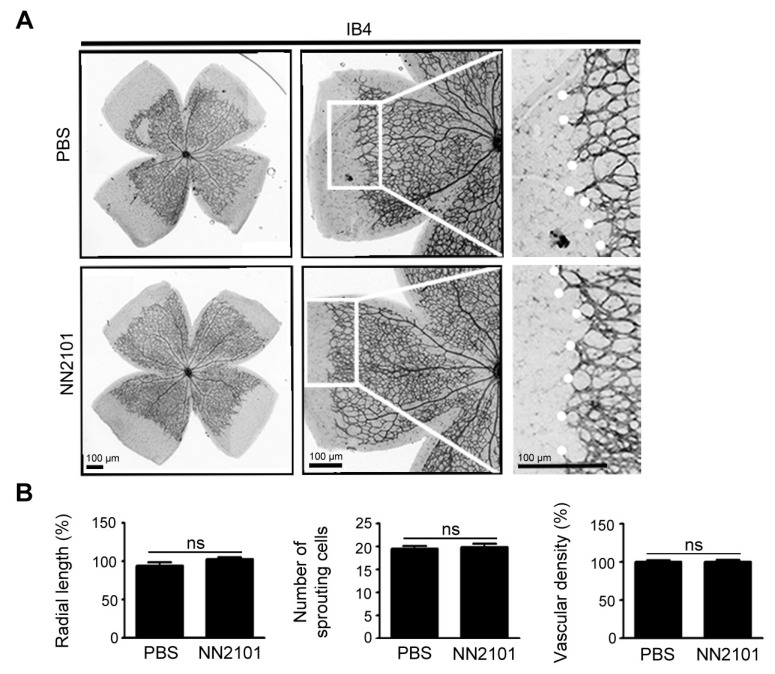
NN2101 does not affect early retinal vascular development in mice. (**A**) representative images of retina vasculature of mice that received an intravitreal injection of NN2101 (20 μg) or PBS at postnatal day (P) 2. Mice were maintained under normoxia until P6, and the retinal vasculature was visualized by staining with IB4 (black). High-magnification views of the growth fronts of vessels are shown on the right and vascular sprouts are marked with white dots. Scale bars = 100 μm; (**B**) quantification of the radial length of blood vessels, number of sprouting endothelial cells, and vascular density in the whole-mounted retinas of mice in (**A**). The data are shown as the mean ± SEM (unpaired Student’s *t*-test, ns = not significant, *n* = 4 mice per group).

We evaluated the effect of NN2101 on normal retinal vascular development in mice. Previously, we showed that depletion of SCF using an anti-SCF neutralizing IgG does not delay the superficial retinal vascular development in mice, and *cKit* mutant (*Kit^W-sh/W-sh^*) mice displayed normal retinal vascular development [12]. Accordingly, we found no significant difference between mice treated intravitreally with NN2101 and PBS vehicle, in terms of vascular density, radial length, and number of sprouting cells (Figure 5). These data indicate that NN2101 did not impair the early physiological retinal vascular development in mice.

### 3.6. PK of Intravitreal NN2101 in the Rabbit Eyes and Serum

**Figure 6:**
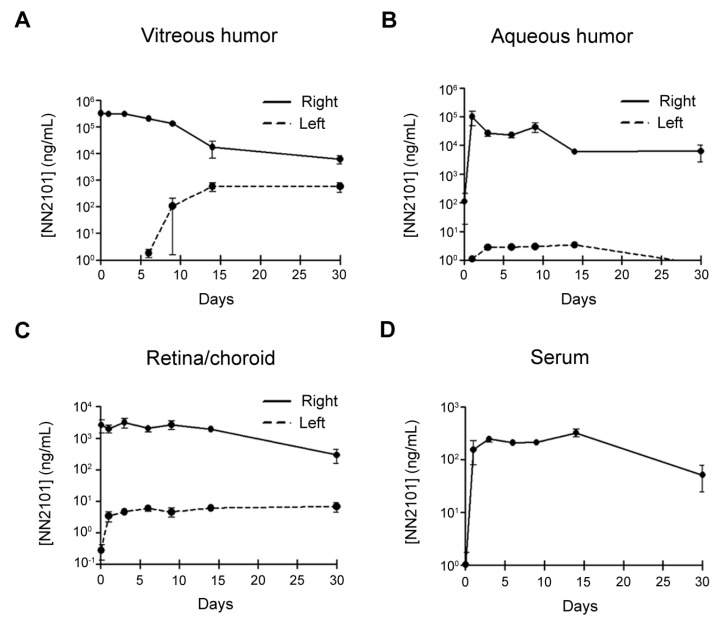
Time-concentration plots for NN2101 in the eyes and serum of rabbits after intravitreal injection. Concentration of NN2101 in vitreous humor (**A**), aqueous humor (**B**), retina and choroid tissues (**C**), and serum (**D**) of rabbits after intravitreal injection of NN2101 (600 μg in 30 μL) is shown. Samples were taken from the injected right (solid line) and uninjected left (dashed line) eyes over 30 days, and the concentration of NN2101 was assessed using indirect ELISA. The data are expressed as the mean ± SEM (*n* = 3 rabbits per time point).

As drugs administered intravitreally should have a long half-life (*T*_1/2_) to avoid frequent administration, we investigated the PK of NN2101 after a single intravitreal injection into rabbits. No adverse events or signs of ocular inflammation were observed after intravitreal administration. The PK parameters of NN2101 in the vitreous humor, aqueous humor, retina/choroid, and blood serum are summarized in Table 1. In the vitreous humor, the concentration of NN2101 peaked 1 h after injection (maximum concentration (C_max_) = 378.3 μg/mL) and declined with a *T*_1/2_ of 103.3 h, which is similar to that of aflibercept (*T*_1/2_ = 94.1 h) (Figure 6A) [19]. Likewise, the mean residence time (MRT) of NN2101 (131.3 h) was comparable to that of aflibercept (135.8 h). Shortly after intravitreal injection, substantial amounts of NN2101 were detected in the aqueous humor and retina/choroid, which suggests that NN2101 in the vitreous humor was rapidly distributed either to the aqueous chamber or to the retina/choroid (Figure 6B,C). The *T*_1/2_ of NN2101 in the aqueous humor and retina/choroid were 203.0 h and 128.9 h, respectively. One day after intravitreal injection, NN2101 reached the maximum serum concentration (C_max_ = 0.3 μg/mL), which was approximately 1200-fold lower than that in the vitreous humor (Figure 6D).

## 4. Discussion

Recently, we reported the novel roles of SCF and cKIT in the regulation of pathological NV in the eyes [12]. Our previous study showed that cKIT and SCF were highly expressed in the angiogenic choroidal vasculature and nearby RPE layers in a murine model of neovascular AMD, and they promoted pathological NV by activating the cKIT/GSK-3β/β-catenin signaling pathway and by enhancing the β-catenin-mediated transcription of the genes related to angiogenesis. These findings prompted us to investigate the potential of a fully human monoclonal IgG targeting cKIT, NN2101, as an alternative or additive therapeutic agent for treating neovascular AMD.

Both in vitro and in vivo experiments confirmed that NN2101 considerably inhibited the activation of SCF/cKIT signaling and reduced the expression of VEGFA, IL-8, and c-Myc, to levels as low as that in normal mice. Intravitreal administration of 1 μg NN2101 significantly decreased the area of CNV lesions with an efficacy comparable to that of the human equivalent dose of aflibercept (2.5 μg). Moreover, the present study revealed that a combination of suboptimal doses of aflibercept and NN2101 inhibited CNV formation with an effect comparable to that of 2.5 μg of aflibercept, which suggested that a combination therapy using aflibercept and NN2101 could help reduce the therapeutic dosages and possible side effects of anti-VEGF agents. NN2101 exhibited no deleterious effect on various human ocular cells. When a high dose of NN2101 was intravitreally administered into normal adult mice, there were no evident changes in the levels of cell apoptosis and retinal tissue stress. Moreover, an intravitreal injection of NN2101 did not affect the early retinal vascular development in mice. These results suggest that anti-SCF/cKIT therapy may efficiently alleviate the pathological CNV without interrupting the normal vascular and neuronal cell homeostasis in the eye. Considering the important role of physiological VEGF in the eye, NN2101 targeting SCF/cKIT signaling would have an advantage over anti-VEGF agents in terms of safety [5,6,7,8,9,10,11].

In the clinical practice of neovascular AMD, an intravitreal injection is the most appropriate administration strategy that can be considered to maximize the therapeutic efficacy of drugs in the posterior segment of the eye and minimize the systemic exposure of drugs [20,21]. Therefore, drugs should present with a long *T*_1/2_ to achieve a sustained therapeutic concentration in the vitreous humor and to reduce the administration frequency. The present data revealed that NN2101 had an adequate PK profile for intravitreal administration. When compared with aflibercept (m.w. = 145 kDa) whose PK data were previously obtained under experimental settings same as that of the present study, NN2101 (m.w. = 180 kDa) exhibited similar PK, including *T*_1/2_, MRT, and apparent clearance in the vitreous humor of a rabbit model. As the vitreous *T*_1/2_ values of aflibercept in humans are estimated to be approximately 2.4–2.8-fold greater than those in rabbits, the *T*_1/2_ of NN2101 in the human vitreous humor might range from 10.3 to 12.1 days, which allows for monthly or bimonthly intravitreal injections during neovascular AMD treatment [19,22,23]. Shortly after an intravitreal injection, a substantial amount of NN2101 was found in the aqueous humor and retina/choroid tissues, which indicated that NN2101 in the vitreous humor was distributed and removed through the anterior and posterior routes. Moreover, NN2101 was detected in the serum one day after intravitreal administration. However, C_max_ and area under the concentration time curve of NN2101 in the serum were approximately 1000-fold and 500-fold lower than those in the vitreous humor, respectively, which suggested that systemic exposure after an intravitreal injection of NN2101 might be too low to interfere with the normal extraocular functions of SCF/cKIT signaling. Nevertheless, further studies are necessary to investigate whether the repeated and long-term administration of NN2101 dysregulates SCF/cKIT signaling and leads to the occurrence of any systemic adverse event in hematopoiesis, fertility, and melanogenesis [24,25,26].

To our knowledge, this is the first study to demonstrate the therapeutic potential of anti-cKIT blocking IgG, NN2101, in the treatment of neovascular AMD. The present study not only revealed that NN2101 suppressed the pathological CNV with a comparable to that of aflibercept, but also suggested that a combination of NN2101 and aflibercept could help confer an additive therapeutic effect on CNV. Moreover, NN2101 showed desirable PK and no ocular toxicity, which is essential for the clinical application of therapeutic IgGs. These findings suggest that NN2101 may be considered a novel promising therapeutic agent for the treatment of neovascular AMD.

## Figures and Tables

**Table 1:** PK parameters of NN2101 in the vitreous humor, aqueous humor, retina/choroid, and serum of rabbits.

PK Parameters	Vitreous	Aqueous	Retina/Choroid	Serum
*T*_1/2_, h	103.3	203.0	128.9	
MRT, h	131.3	208.5	243.7	311.6
V/F, mL	1.3	12.0	98.1	
CL/F, mL/h	0.009	0.041	0.527	
C_max_, μg/mL	378.3	100.8	3.2	0.3
AUC, h × μg/mL	63,921.0	12,771.9	1101.6	132.7

*T*_1/2_, half-life; MRT, mean residence time; V/F, apparent volume of distribution; CL/F, apparent clearance; C_max_, maximum concentration; AUC, area under the concentration time curve.

## Data Availability

The data presented in this study are available from the corresponding author on reasonable request.

## Supplementary Materials

The following are available online at https://www.mdpi.com/article/10.3390/pharmaceutics13081308/s1, Figure S1: NN2101 effectively inhibits SCF-mediated in vitro angiogenesis in murine endothelial cells at hypoxia; Table S1: IgGs used for Western blotting and immunohistochemistry.

## References

[1] Wong W.L., Su X., Li X., Cheung C.M., Klein R., Cheng C.Y., Wong T.Y. (2014). Global prevalence of age-related macular degeneration and disease burden projection for 2020 and 2040: A systematic review and meta-analysis. Lancet Glob. Health.

[2] Grisanti S., Tatar O. (2008). The role of vascular endothelial growth factor and other endogenous interplayers in age-related macular degeneration. Prog. Retin. Eye Res..

[3] Campochiaro P.A. (2015). Molecular pathogenesis of retinal and choroidal vascular diseases. Prog. Retin. Eye Res..

[4] Holz F.G., Schmitz-Valckenberg S., Fleckenstein M. (2014). Recent developments in the treatment of age-related macular degeneration. J. Clin. Investig..

[5] Bhisitkul R.B., Mendes T.S., Rofagha S., Enanoria W., Boyer D.S., Sadda S.R., Zhang K. (2015). Macular atrophy progression and 7-year vision outcomes in subjects from the ANCHOR, MARINA, and HORIZON studies: The SEVEN-UP study. Am. J. Ophthalmol..

[6] Ford K.M., Saint-Geniez M., Walshe T., Zahr A., D’Amore P.A. (2011). Expression and role of VEGF in the adult retinal pigment epithelium. Investig. Ophthalmol. Vis. Sci..

[7] Grunwald J.E., Pistilli M., Daniel E., Ying G.S., Pan W., Jaffe G.J., Toth C.A., Hagstrom S.A., Maguire M.G., Martin D.F. (2017). Incidence and Growth of Geographic Atrophy during 5 Years of Comparison of Age-Related Macular Degeneration Treatments Trials. Ophthalmology.

[8] Kurihara T., Westenskow P.D., Bravo S., Aguilar E., Friedlander M. (2012). Targeted deletion of Vegfa in adult mice induces vision loss. J. Clin. Investig..

[9] Sadda S.R., Tuomi L.L., Ding B., Fung A.E., Hopkins J.J. (2018). Macular Atrophy in the HARBOR Study for Neovascular Age-Related Macular Degeneration. Ophthalmology.

[10] Spitzer M.S., Wallenfels-Thilo B., Sierra A., Yoeruek E., Peters S., Henke-Fahle S., Bartz-Schmidt K.U., Szurman P., Tuebingen Bevacizumab Study G. (2006). Antiproliferative and cytotoxic properties of bevacizumab on different ocular cells. Br. J. Ophthalmol..

[11] Usui-Ouchi A., Friedlander M. (2019). Anti-VEGF therapy: Higher potency and long-lasting antagonism are not necessarily better. J. Clin. Investig..

[12] Kim K.L., Seo S., Kim J.T., Kim J., Kim W., Yeo Y., Sung J.H., Park S.G., Suh W. (2019). SCF (Stem Cell Factor) and cKIT Modulate Pathological Ocular Neovascularization. Arter. Thromb. Vasc. Biol..

[13] Abu El-Asrar A.M., Nawaz M.I., Kangave D., Mairaj Siddiquei M., Geboes K. (2013). Angiogenic and vasculogenic factors in the vitreous from patients with proliferative diabetic retinopathy. J. Diabetes Res..

[14] Abu El-Asrar A.M., Struyf S., Opdenakker G., Van Damme J., Geboes K. (2010). Expression of stem cell factor/c-kit signaling pathway components in diabetic fibrovascular epiretinal membranes. Mol. Vis..

[15] Kim J.O., Kim H.N., Kim K.H., Baek E.J., Park J.Y., Ha K., Heo D.R., Seo M.D., Park S.G. (2020). Development and characterization of a fully human antibody targeting SCF/c-kit signaling. Int. J. Biol. Macromol..

[16] Kim K.L., Suh W. (2017). Apatinib, an Inhibitor of Vascular Endothelial Growth Factor Receptor 2, Suppresses Pathologic Ocular Neovascularization in Mice. Investig. Ophthalmol. Vis. Sci..

[17] Lambert V., Lecomte J., Hansen S., Blacher S., Gonzalez M.L., Struman I., Sounni N.E., Rozet E., de Tullio P., Foidart J.M. (2013). Laser-induced choroidal neovascularization model to study age-related macular degeneration in mice. Nat. Protoc..

[18] Chou T.C. (2006). Theoretical basis, experimental design, and computerized simulation of synergism and antagonism in drug combination studies. Pharm. Rev..

[19] Park S.J., Choi Y., Na Y.M., Hong H.K., Park J.Y., Park K.H., Chung J.Y., Woo S.J. (2016). Intraocular Pharmacokinetics of Intravitreal Aflibercept (Eylea) in a Rabbit Model. Investig. Ophthalmol. Vis. Sci..

[20] Meyer C.H., Krohne T.U., Charbel Issa P., Liu Z., Holz F.G. (2016). Routes for Drug Delivery to the Eye and Retina: Intravitreal Injections. Dev. Ophthalmol..

[21] Varela-Fernandez R., Diaz-Tome V., Luaces-Rodriguez A., Conde-Penedo A., Garcia-Otero X., Luzardo-Alvarez A., Fernandez-Ferreiro A., Otero-Espinar F.J. (2020). Drug Delivery to the Posterior Segment of the Eye: Biopharmaceutic and Pharmacokinetic Considerations. Pharmaceutics.

[22] Christoforidis J.B., Williams M.M., Kothandaraman S., Kumar K., Epitropoulos F.J., Knopp M.V. (2012). Pharmacokinetic properties of intravitreal I-124-aflibercept in a rabbit model using PET/CT. Curr. Eye Res..

[23] Garcia-Quintanilla L., Luaces-Rodriguez A., Gil-Martinez M., Mondelo-Garcia C., Maronas O., Mangas-Sanjuan V., Gonzalez-Barcia M., Zarra-Ferro I., Aguiar P., Otero-Espinar F.J. (2019). Pharmacokinetics of Intravitreal Anti-VEGF Drugs in Age-Related Macular Degeneration. Pharmaceutics.

[24] Broudy V.C. (1997). Stem cell factor and hematopoiesis. Blood.

[25] Sette C., Dolci S., Geremia R., Rossi P. (2000). The role of stem cell factor and of alternative c-kit gene products in the establishment, maintenance and function of germ cells. Int. J. Dev. Biol..

[26] Yoshida H., Kunisada T., Grimm T., Nishimura E.K., Nishioka E., Nishikawa S.I. (2001). Review: Melanocyte migration and survival controlled by SCF/c-kit expression. J. Investig. Dermatol. Symp. Proc..

