# Supplementary material for: A Fully Human Monoclonal Antibody Targeting cKIT Is a Potent Inhibitor of Pathological Choroidal Neovascularization in Mice
Source: Pharmaceutics. 2021 Aug 20;13(8):1308. doi: 10.3390/pharmaceutics13081308 (PMC8400730; doi:10.3390/pharmaceutics13081308)
Supplement: Supplementary file 1 [file pharmaceutics-13-01308-s001.zip › pharmaceutics-1313278-supplementary.pdf]

pharmaceutics

MDPI

Supplementary Materials: A Fully Human Monoclonal Antibody Targeting cKIT Is a Potent Inhibitor of Pathological Choroidal Neovascularization in Mice

Songyi Seo, Koung Li Kim, Yeongju Yeo, Ryul-I Kim, Hayoung Jeong, Jin-Ock Kim, Sun-Hwa Song, Mi-Jin An, Jung-Woong Kim, Hye Kyoung Hong, Min Hee Ham, Se Joon Woo, Jong-Hyuk Sung, Sang Gyu Park and Wonhee Suh

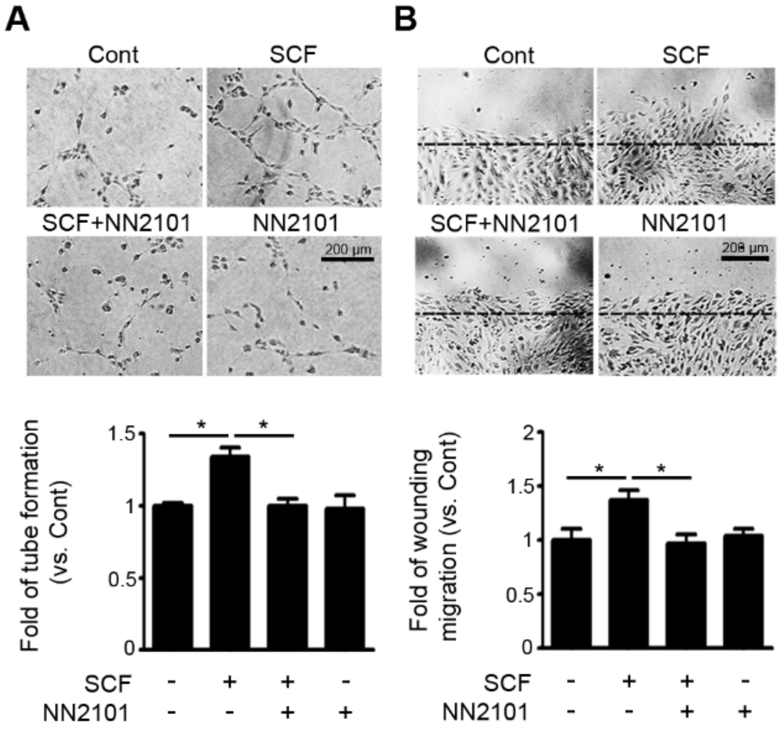

Figure S1. NN2101 effectively inhibits SCF-mediated *in vitro* angiogenesis in murine endothelial cells at hypoxia. (**A**) Tube formation and (**B**) scratch wounding migration assays were performed using murine endothelial cells subjected to treatment with or without recombinant mouse SCF (50 ng/mL) and NN2101 (1 µg/mL) at hypoxia. Tube formation and scratch wound migration results were quantified by measuring the tube length and relative area covered by cells that had migrated from the wound edges, respectively. The data are expressed as the fold increase ± SEM compared to the untreated control group (one-way ANOVA with Bonferroni *post hoc* multiple comparison test, *$p < 0.05$, $n > 2$).

Table S1. IgGs used for western blotting and immunohistochemistry.

IgG	Supplier	Catalog Number	Dilution Ratio
p-cKIT (Tyr719)	Cell Signaling Technology (Danvers, MA)	3391	1:1000
cKIT (human)	R&D Systems (Minneapolis, MN)	AF332	1:1000
cKIT (Mouse)	R&D Systems	MAB1356	1:1000
p-AKT (ser473)	Cell Signaling Technology	9271	1:1000
AKT	Cell Signaling Technology	9272	1:1000
p-GSK-3β (Ser9)	Cell Signaling Technology	9323	1:1000
GSK-3β	Cell Signaling Technology	9315	1:1000
p-β-catenin (Ser33/37/Thr41)	Cell Signaling Technology	9561	1:1000
β-catenin	BD Biosciences (San Diego, CA)	610153	1:1000
PARP	Santa Cruz Biotechnology (Santa Cruz, CA)	SC8007	1:1000
β-actin (human)	Sigma-Aldrich (St. Louis, MO)	A2066	1:2500
β-actin (mouse)	Santa Cruz Biotechnology	SC-47778	1:2500
VEGFA (human)	Abcam (Cambridge, UK)	AB4615	1:1000
VEGFA (mouse)	Santa Cruz Biotechnology	SC-152	1:1000
IL-8 (human)	Abcam	AB235584	1:1000
IL-8 (mouse)	Abcam	AB183705	1:1000
c-Myc	Cell Signaling Technology	13987	1:1000
CyclinD1	Cell Signaling Technology	2976	1:1000
Peroxidase-labeled anti-goat IgG	VECTOR (Burlingame, CA)	PI-9500	1:2000
Peroxidase-labeled anti-mouse IgG	VECTOR	PI-2000	1:2000
Peroxidase-labeled anti-rabbit IgG	VECTOR	PI-1000	1:2000
GFAP	Dako (Carpinteria, CA)	Z0334	1:1000
